# Prevalence and predictors of tuberculosis infection among people living with HIV in a high tuberculosis burden context

**DOI:** 10.1136/bmjresp-2022-001581

**Published:** 2023-05-17

**Authors:** Lilian Nkirote Njagi, Videlis Nduba, Marianne Wanjiru Mureithi, Jared Ongechi Mecha

**Affiliations:** 1 Center for Respiratory Disease Research, Kenya Medical Research Institute, Nairobi, Kenya; 2 Department of Medical Microbiology & Immunology, University of Nairobi Faculty of Health Sciences, Nairobi, Kenya; 3 Department of Clinical Medicine and Therapeutics, University of Nairobi Faculty of Health Sciences, Nairobi, Kenya

**Keywords:** Immunodeficiency, Tuberculosis, Clinical Epidemiology

## Abstract

**Background:**

Tuberculosis (TB) disease is the leading cause of mortality among people living with HIV (PLHIV). Interferon-gamma release assays (IGRAs) are approved for TB infection ascertainment. However, current IGRA data on the prevalence of TB infection in the context of near-universal access to antiretroviral therapy (ART) and TB preventive therapy (TPT) are lacking. We estimated the prevalence and determinants of TB infection among PLHIV within a high TB and HIV burden context.

**Methods:**

This cross-sectional study included data from adult PLHIV age ≥18 years in whom QuantiFERON-TB Gold Plus (QFT-Plus) assay, an IGRA, was performed. TB infection was defined as a positive or indeterminate QFT-Plus test. Participants with TB and those who had previously used TPT were excluded. Regression analysis was performed to identify independent predictors of TB infection.

**Results:**

Of 121 PLHIV with QFT-Plus test results, females were 74.4% (90/121), and the mean age was 38.4 (SD 10.8) years. Overall, 47.9% (58/121) were classified as TB infection (QFT-Plus test positive and indeterminate results were 39.7% (48/121) and 8.3% (10/121), respectively). Being obese/overweight (body mass index ≥25 kg/m^2^; p=0.013, adjusted OR (aOR) 2.90, 95% CI 1.25 to 6.74) and ART usage for >3 years (p=0.013, aOR 3.99, 95% CI 1.55 to 10.28) were independently associated with TB infection.

**Conclusion:**

There was a high TB infection prevalence among PLHIV. A longer period of ART and obesity were independently associated with TB infection. The relationship between obesity/overweight and TB infection may be related to ART use and immune reconstitution and requires further investigation. Given the known benefit of test-directed TPT among PLHIV never exposed to TPT, its clinical and cost implications for low and middle-income countries should be explored further.

**WHAT IS ALREADY KNOWN ON THIS TOPIC**

- Among people living with HIV (PLHIV), the risk of progression to tuberculosis (TB) disease is higher with confirmed and untreated TB infection. Data on the prevalence of TB infection in the context of near-universal access to antiretroviral therapy (ART) and TB preventive therapy (TPT) are lacking in Africa.

**WHAT THIS STUDY ADDS**

- Using the QuantiFERON-TB Gold Plus assay for diagnosis, this study provides evidence that the prevalence of TB infection remains high even with near-universal access to ART and TPT.

**HOW THIS STUDY MIGHT AFFECT RESEARCH, PRACTICE OR POLICY**

- This study should prompt larger studies to explore the prevalence and evaluate determinants of TB infection among PLHIV. A broader understanding of the clinical and cost implications of test-directed TPT for PLHIV in low and middle-income countries may better inform policy towards its utility.

## Introduction

Tuberculosis (TB) continues to be a global health concern.^1–3^ Despite near-universal access to antiretroviral therapy (ART) and TB preventive therapy (TPT), TB remains the leading cause of disease and mortality among people living with HIV (PLHIV).^3–5^ In 2021, approximately 10.6 million people developed TB worldwide, 6.7% (703 000) of whom were PLHIV.^3 6^ In the same period, 187 000 PLHIV died from TB, accounting for one-third of all AIDS-related deaths globally.^3 5^ Kenya is among the 30 high-burden countries for TB and HIV-associated TB.^3^ In 2021, the country had 133 000 incident TB cases, and 24% (32 000) were HIV/TB coinfected, of whom 34% (11 000) died.^6^ TB infection, the precursor of TB disease, is a crucial focus for TB prevention strategies.^3 7–9^ The global burden of TB disease is estimated at a quarter of the population,^10^ with wide regional variation.^11 12^

Current information on the burden of TB infection in Africa and among PLHIV is sparse.^11 12^ There are also limited data on how the prevalence of TB infection has changed with near-universal access to ART and TPT. Other factors associated with TB infection among PLHIV are underexplored.^13–15^ Establishing the burden of TB infection and the associated risk factors in PLHIV is important for various reasons. First, the risk of progression from TB infection to TB disease is higher among PLHIV with a positive test for TB infection than among those with a negative or unknown test result.^16^ Second, TB risk is markedly reduced by TPT, with a 64% risk reduction among PLHIV with a positive test, compared with 14% among those with a negative or unknown test.^17^ This has potential implications for practice, given the WHO and national recommendation to treat all PLHIV for TB infection without the need for confirmatory testing.^9 18–20^

The use of gold-standard tests to establish disease burden is optimum when feasible.^21 22^ No gold standard exists for the diagnosis of TB infection. However, interferon-gamma release assays (IGRAs) and tuberculin skin tests are screening tests approved by the WHO.^9^ IGRAs are advantageous since prior BCG vaccination does not affect their performance characteristics.^23^ HIV impacts the QuantiFERON-TB Gold In-Tube test (QFT-GIT) less than it does the ELISPOT (T SPOT-TB).^24 25^ The QFT Gold Plus (QFT-Plus) and its forerunner, the QFT-GIT, have concordant performance.^26 27^ This paper presents the results of a study that sought to estimate TB infection prevalence among PLHIV using the QFT-Plus assay in a high TB burden context. Further, the determinants of TB infection in this population were explored.

## Methods

### Study setting and design

This cross-sectional study used data collected at enrolment into a prospective observational study to explore methods of monitoring response to isoniazid during TPT. The study was conducted at three HIV care and prevention centres in Nairobi, Kenya comprising Kenyatta National Hospital, Kenya’s largest national teaching and referral hospital; Pumwani maternity hospital, Kenya’s largest referral maternity hospital and the Kenya Medical Research Institute, Centre for Respiratory disease Research. The clientele is cosmopolitan, ranging from urban, periurban and rural settings.

### Study period and population

**Figure 1:**
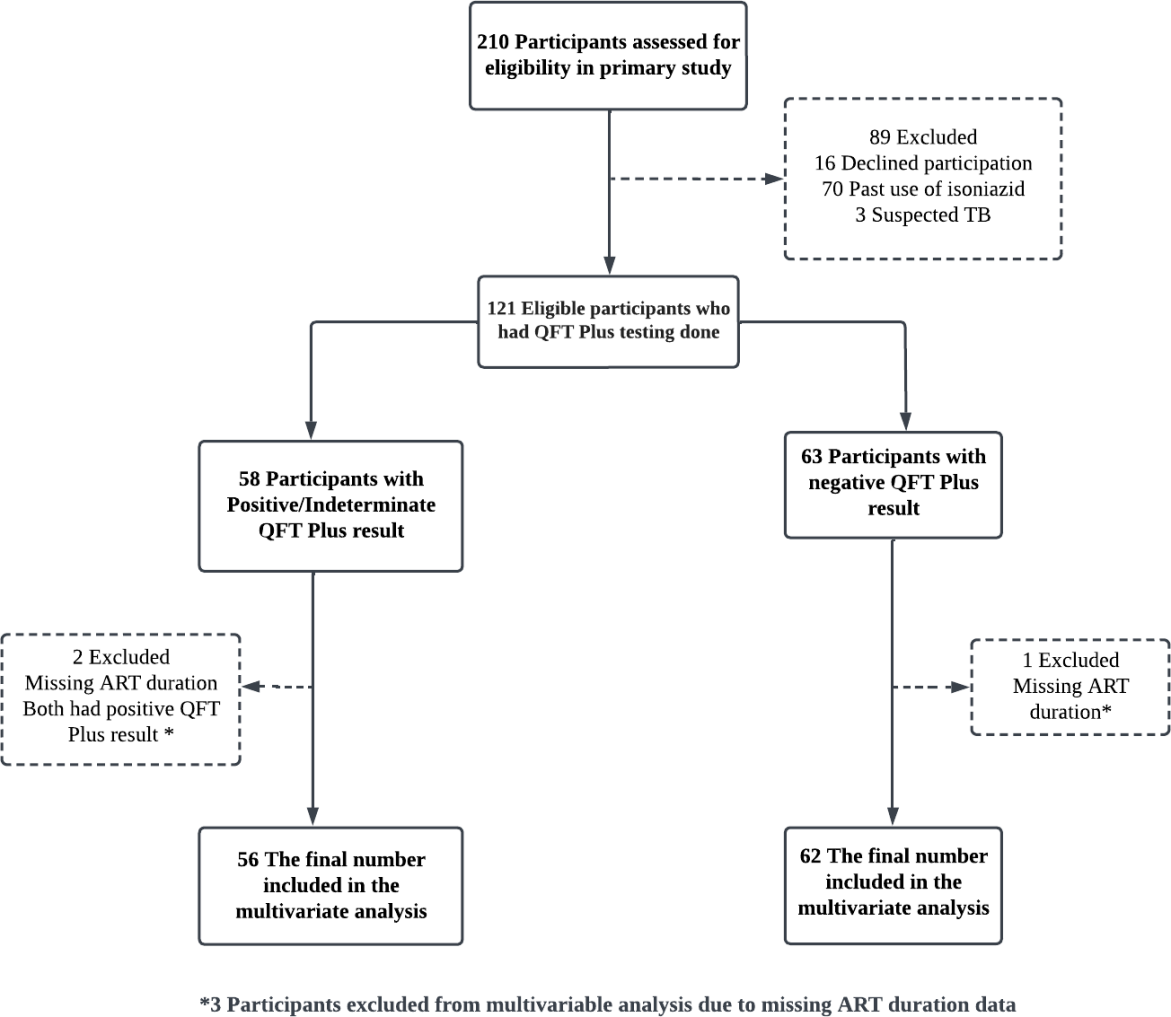
Enrolment and analysis flow diagram. ART, antiretroviral therapy; QFT, QuantiFERON-TB; TB, tuberculosis.

Participants aged ≥18 years seeking HIV care and prevention services between December 2019 and December 2020 were eligible to participate in the primary study if verbal and written informed consent was obtained. To reduce the chance of outcome misclassification, participants who had ever received isoniazid as a treatment for TB infection or disease and those suspected or confirmed as having TB disease were ineligible. All study participants underwent the recommended four-item TB symptom screening questions to rule out TB disease.^28^ Any participants with an affirmative response to any of the four questions received further evaluation, including a complete physical examination, sputum examination for cartridge-based nucleic acid amplification test and chest X-ray. This cross-sectional study includes all the 121 PLHIV that met the eligibility criteria for the primary study (figure 1).

### Variables and definitions

The outcome variable of interest was the TB infection status. TB infection was defined as a positive or indeterminate QFT-Plus result. We included indeterminate results in the definition of TB infection for several reasons. Foremost, we were bound by the existing WHO and national recommendations to treat all PLHIV for TB infection without requiring testing.^9 18 20 28^ Further, guidance on handling indeterminate QFT-Plus tests among PLHIV in the context of TPT is lacking. The WHO recommends a ‘case-by-case assessment for the potential benefit and harms of TPT’ in regard to general testing for TB infection.^9^ Second, the manufacturer suggests repeat testing for indeterminate results when related to technical factors where instructions are not followed.^29^ In our case, instructions were followed per protocol. Finally, as this was a cross-sectional study and resources were limited, prospective repeat testing was not feasible, and clinical care took precedence over repeat testing.^30 31^ All participants with negative results were considered not to have TB infection. The explanatory variables of interest were age, sex, body mass index (BMI), cigarette smoking, alcohol use, household crowding, history of contact with a known TB case, diabetes status, duration of ART use as ≤3 vs >3 years and viral load level as ≤40 vs >40 HIV copies/mL of blood. To measure smoking and alcohol use habits, participants were asked if they had ever smoked and if they considered their alcohol use more than social. Similarly, participants were asked whether their household was crowded, whether they had been in contact with a person diagnosed with TB in the preceding 2 years, and if they had ever been diagnosed with diabetes.

### Clinical and laboratory procedures

At baseline, clinical and demographic data were collected or abstracted from electronic medical records. These included age, sex, weight, height, smoking status, alcohol use, living conditions, diabetes status and other known chronic illness, history of TB infection and isoniazid use. HIV rapid testing was performed in programmatic settings for those of unknown status as part of routine HIV prevention. Where feasible, a CD4 lymphocyte count was performed. Viral load testing was performed under programmatic settings per the existing national guidelines. All participants on ART for at least 6 months had viral load testing done at the same time as the QFT-Plus test. All participants that had been on ART for less than 6 months at the time of the QFT-Plus test had viral load done after 6 months of ART use according to national guidelines. The ART regimen and initiation date were collected, and the date was used to determine the duration of ART use. Viral load level (≤40 vs >40 copies/mL) and ART duration (≤3 vs >3 years) were used as a proxy for immune reconstitution and competency in the absence of CD4 lymphocyte count.

We used the QFT-Plus assay for TB infection diagnosis according to the manufacturer’s protocol.^29^ Briefly, 6 mL of peripheral venous whole blood was collected into lithium heparin vacutainers and transported to the laboratory within 2 hours of collection. A 1 mL was transferred into the four QFT-Plus blood collection tubes, the Nil tube, TB1 tube, TB2 tube and the mitogen tube within 8 hours of collection and incubated at 37°C for 16 hours. The tubes were centrifuged for 15 min at 2000–3000×g RCF (g). Plasma was collected and assessed using the standard ELISA.

The results were interpreted according to the manufacturer’s instructions.^29^ A QFT-Plus result was interpreted as positive when the IFN-γ response to one or both MTB-specific antigens was ≥0.35 IU/mL and ≥25% of Nil value, irrespective of the IFN-γ response to the mitogen control. A QFT-Plus result was interpreted as negative when the IFN-γ response to both MTB-specific proteins was <0.35 IU/mL, or≥0.35 IU/mL and <25% of Nil value, with a response to the mitogen control ≥0.5 IU/mL. A QFT-Plus result was interpreted as indeterminate when the IFN-γ response to both MTB-specific proteins was <0.35 IU/mL, or ≥0.35 IU/mL and <25% of Nil value, with a response to the mitogen control <0.5 IU/mL. A QFT-Plus result was interpreted as indeterminate when the IFN-γ response was above the cut-off in the nil control, irrespective of the IFN-γ response to the MTB-specific antigens and the mitogen control.^29^

For quality control, independent study staff checked all consent forms, study questionnaires and laboratory results for accuracy and completeness on the same day of data entry.

### Sample size

The sample size was determined based on an estimated TB infection prevalence of 35% in exposed^32^ and 10% in unexposed^10^ at a 95% level of confidence and 80% power. Thus, a sample of 102 with continuity correction was sufficient, and all 121 eligible participants that were HIV-infected and that consented were selected for the study.

### Statistical analysis

After appropriate data cleaning, the analysis was performed using Stata Statistical Software V.17 (StataCorp). Descriptive statistics were used to summarise the sociodemographic and clinical characteristics of the participants. Pearson’s χ^2^ and Fisher’s exact tests were used to test for the association between categorical variables and outcomes, and the ORs were reported. For continuous variables, the Student’s t-test was used. The multivariate logistic regression analyses included factors with a p<0.10. Statistical significance was set at p<0.05. Missing data were excluded to reduce the chance of biased estimates. Specifically, CD4 lymphocyte count data were excluded from the analysis and substituted with the duration of ART use. Complete case analysis was performed during multivariate regression (figure 1).

### Patient and public involvement

It was not possible to involve patients or the public in the design, conduct, reporting or dissemination plans of the study. Patients were involved to the extent to which they provided informed consent and allowed for sample collection for laboratory testing. The study results will be communicated to willing participants and community members.

## Results

### Sociodemographic and clinical characteristics of participants

Among the 121 study participants living with HIV included in this analysis, females were the majority at 74.4% (90/121). The mean age of all participants was 38.4 years with a standard deviation (SD) of 10.8, and 51.2% (62/121) of the participants were ≥40 years. By BMI, 15.7% (19/121) of the participants were categorised as obese, 30.6% (37/121) as overweight, 49.6% (60/121) as normal and 4.1% (5/121) as underweight. Among the social risk factors, 18.2% (22/121) were cigarette smokers, 20.7% (25/121) used alcohol, 27.3% (33/121) reported living in a crowded place and 26.4% (32/121) had known contact with TB cases. Regarding clinical and laboratory characteristics, only 2.5% (3/121) had diabetes, and 38.1% (45 of 118 with complete ART duration data) had been on ART for >3 years. All participants were on the recommended first-line regimens with a backbone of tenofovir and lamivudine, combined with dolutegravir in 95% (115) and efavirenz in the remaining 5%. The participants with viral load <40 copies/mL were 85.7% (102 of 119 with complete viral load data). Table 1 shows the participants’ sociodemographic and clinical characteristics.

**Table 1:** Sociodemographic and clinical characteristics of participants (n=121)

Variable	Frequency (n=121)	No TB infection* (n=63)	TB infection† (n=58)
Demographic variables			
Gender (n, %)			
Male	31	16 (51.6)	15 (48.4)
Female	90	47 (52.2)	43 (47.8)
Age (mean, SD)	38.4, 10.8	(38.2, 11.3)	(38.7, 10.3)
Age groups (n, %)			
<30	26	15 (57.7)	11 (42.3)
30–39	33	19 (57.6)	14 (42.4)
≥40	62	29 (46.8)	33 (53.2)
BMI (n, %)			
<18.5	5	3 (60.0)	2 (40.0)
18.5 to <25.0	60	37 (61.7)	23 (38.3)
25.0 to 30.0	37	12 (32.4)	25 (67.6)
≥30	19	11 (57.9)	8 (42.1)
Social risk factors			
History of smoking (n, %)			
No	99	57 (57.6)	42 (42.4)
Yes	22	6 (27.3)	16 (72.7)
Alcohol use (n, %)			
No	96	54 (56.3)	42 (43.8)
Yes	25	9 (36.0)	16 (64.0)
Household crowding (n, %)			
Not crowded	88	47 (53.4)	41 (46.6)
Crowded	33	16 (48.5)	17 (51.5)
History of contact with TB case (n, %)			
No	89	51 (57.3)	38 (42.7)
Yes	32	12 (37.5)	20 (62.5)
Clinical and laboratory characteristics			
Diabetes status (n, %)			
No	118	60 (50.9)	58 (49.2)
Yes	3	3 (100.0)	0 (0.0)
Duration of ART use (n, %)‡			
≤3 years	73	45 (61.6)	28 (38.4)
>3 years	45	17 (37.8)	28 (62.2)
Viral load level (copies/mL; n, %)§			
≤40	102	52 (51.0)	50 (49.0)
>40	17	9 (52.9)	8 (47.1)

*Negative QFT-Plus test.

†Positive and indeterminate QFT-Plus test.

‡n=118, 3 participants did not have a documented ART start date.

§n=119, 2 participants did not have a valid viral load report.

ART, antiretroviral therapy; BMI, body mass index; QFT, QuantiFERON-TB; TB, tuberculosis.

### Prevalence of TB infection estimated by QFT-Plus test results

The prevalence of TB infection estimated by QFT-Plus test results in this population of PLHIV was 47.9% (58/121; table 1). This comprised 39.7% (48/121) with a positive QFT-Plus test report and 8.3% (10/121) with an indeterminate QFT-Plus test report. Participants with an indeterminate QFT-Plus are described in table 2. Among the participants with indeterminate results, 60% (6/10) were female, and 80% (8/10) were ≥30 years old. All the participants reported having HIV as the only underlying chronic illness known to them. Only 30% (3/10) reported cigarette use, and only 40% (4/10) reported alcohol use. Sixty per cent (6/10) of participants had been on ART for less than 1 year. In 8 of the 10 participants with indeterminate results, the interferon-gamma (IFN-γ) level in response to mitogen was below the cut-off of 0.5 IU/mL for mitogen minus Nil (table 2). Three of the 10 participants had the IFN-γ response in the Nil tube above the cut-off of 8.0 IU/mL (table 2), signifying an ongoing immune response.

**Table 2:** Characteristics of participants with indeterminate QFT-Plus test results (n=10)

Patient	Age	Sex	Duration on ART (years)	BMI	History of smoking	Alcohol use	IFN-level (IU/mL)			
							Nil	TB1-Nil	TB2-Nil	Mitogen-Nil
1	30–39	F	<1	25	No	Yes	0.08	−0.03	−0.03	0.02
2	30–39	F	<1	16	Yes	No	>10	>10	>10	>10
3	30–39	F	<1	18	Yes	Yes	>10	−15.63	−59.3	−66.84
4	<30	M	<1	22	No	Yes	0.09	0.04	−0.03	0.04
5	≥40	F	11	24	No	No	0.62	−0.34	−0.36	0.15
6	≥40	M	11	27	No	No	0.09	0.06	−0.02	0.14
7	≥40	F	4	30	No	No	>10	>10	>10	>10
8	30–39	F	<1	26	No	No	0.06	0	−0.01	0.11
9	<30	M	<1	27	Yes	Yes	1.18	−0.16	−0.35	0.02
10	≥40	M	11	21	No	No	0.07	−0.04	−0.02	0.18

ART, antiretroviral therapy; BMI, body mass index; F, female; M, male; QFT, QuantiFERON-TB.

### Determinants of TB infection estimated by QFT-Plus test results

The mean age of participants with TB infection was 38.7 years (SD 10.3) compared with 38.2 years (SD 11.3) among those without TB infection (p=0.6022) (table 3). On bivariate analysis, increased odds of having TB infection were observed among participants aged ≥40 years (p=0.353, OR 1.55, 95% CI 0.61 to 3.95), those with reported alcohol use (p=0.071, OR 2.29, 95% CI 0.90 to 5.78), those living in a crowded place (p=0.631, OR 1.22, 95% CI 0.54 to 2.72) and those with history of contact with a case of TB (p=0.056, OR 2.24, 95% CI 0.96 to 5.21). However, these findings were not statistically significant at p<0.05 (table 3). BMI (p=0.025, OR 2.30, 95% CI 1.09 to 4.85), a history of smoking cigarettes (p=0.010, OR 3.62, 95% CI 1.26 to 10.37) and duration of ART use >3 years (p=0.012, OR 2.65, 95% CI 1.20 to 5.83) were significantly associated with TB infection (table 3).

**Table 3:** Determinants of TB infection among PLHIV—bivariate analysis (n=121)

Variable	No TB infection* (n=63)	TB infection† (n=58)	P value	OR‡ (95% CI)	χ^2^
Baseline characteristics					
Gender (n, %)					
Male Female	16 (51.6) 47 (52.2)	15 (48.4) 43 (47.8)	0.953	Ref 0.98 (0.43 to 2.22)	0.00
Age (mean, SD)	(38.2, 11.3)	(38.7, 10.3)	0.602	–	–
Age groups (n, %)					
<30 30–39 ≥40	15 (57.7) 19 (57.6) 29 (46.8)	11 (42.3) 14 (42.4) 33 (53.2)	Ref 0.993 0.353	Ref 1.00 (0.35 to 2.87) 1.55 (0.61 to 3.95)	1.43
BMI (n, %)					
<25 ≥25.0	40 (61.5) 23 (41.1)	25 (38.5) 33 (58.9)	**0.025**	Ref 2.30 (1.09 to 4.85)	5.01
Social risk factors					
History of smoking (n, %)					
No Yes	57 (57.6) 6 (27.3)	42 (42.4) 16 (72.7)	**0.010**	Ref 3.62 (1.26 to 10.37)	6.57
Alcohol use (n, %)					
No Yes	54 (56.3) 9 (36.0)	42 (43.8) 16 (64.0)	**0.071**	Ref 2.29 (0.90 to 5.78)	3.23
Household crowding (n, %)					
Not crowded Crowded	47 (53.4) 16 (48.5)	41 (46.6) 17 (51.5)	0.631	Ref 1.22 (0.54 to 2.72)	0.23
Contact with TB§ case (n, %)					
No Yes	51 (57.3) 12 (37.5)	38 (42.7) 20 (62.5)	**0.056**	Ref 2.24 (0.96 to 5.21)	3.67
Clinical and laboratory characteristics					
Diabetes status (n, %)					
No Yes	60 (50.9) 3 (100.0)	58 (49.2) 0 (0.0)	0.094	Ref 0.00(-)	2.81
Duration of ART use (n, %)¶					
≤3 years >3 years	45 (61.6) 17 (37.8)	28 (38.4) 28 (62.2)	**0.012**	Ref 2.65 (1.20 to 5.83)	6.31
Viral load level (copies/ml; n, %)**					
≤40 >40	52 (51.0) 9 (52.9)	50 (49.0) 8 (47.1)	0.881	Ref 0.92 (0.33 to 2.60)	0.02

The bold values are factors with a p<0.10 included in multivariate analyses.

*Negative QFT-Plus test.

†Positive & indeterminate QFT-Plus test.

‡Odds ratio.

§Tuberculosis.

¶n=118, 3 participants did not have a documented ART start date.

**n=119, 2 participants did not have a valid viral load report.

ART, antiretroviral therapy; BMI, body mass index; TB, tuberculosis.

In a subanalysis, we found that among factors associated with being on ART>3 years were age >40 years old (p=0.005, OR 4.57, 95% CI 1.42 to 14.7) and having a positive QFT-Plus test (p=0.009, OR 2.89, 95% CI 1.25 to 6.65). Although increased odds of being on ART>3 years were observed among participants with indeterminate results (p=0.420, OR 1.76, 95% CI 0.44 to 7.15), this finding was not statistically significant. Reduced odds of being on ART>3 years were observed among participants having a viral load of >40 copies/mL (p=0.772, OR 0.85, 95% CI 0.29 to 2.51) (online supplemental table 1).

10.1136/bmjresp-2022-001581.supp1 Supplementary data

On multivariate analysis including factors with p<0.10 (table 4), there were increased odds of TB infection among those using alcohol (p=0.066, adjusted OR (aOR) 2.88, 95% CI 0.93 to 8.91). Being obese/overweight (BMI≥25 kg/m^2^; p=0.013, aOR 2.90, 95% CI 1.25 to 6.74) and being on ART for >3 years (p=0.013, aOR 3.99, 95% CI 1.55 to 10.28) were independently associated with TB infection (table 4). We performed sensitivity analyses in which we excluded the category with BMI<18.5 kg/m^2^ (online supplemental table 2) and again considered indeterminate QFT-Plus tests as negative (online supplemental table 3). The observed associations remained.

10.1136/bmjresp-2022-001581.supp2 Supplementary data

10.1136/bmjresp-2022-001581.supp3 Supplementary data

**Table 4:** Logistic regression analysis for factors associated with TB infection (n=118)

Variable	No TB infection* (n=62)	TB infection† (n=56)	P value	cOR‡ (95% CI)	aOR§ (95% CI)
Baseline characteristics					
BMI (n, %)					
<25 ≥25.0	39 (61.9) 23 (41.8)	24 (38.1) 32 (58.2)	**0.013**	Ref 2.26 (1.08 to 4.73)	Ref 2.90 (1.25 to 6.74)
Social risk factors					
History of smoking (n, %)					
No Yes	56 (57.7) 6 (28.6)	41 (42.3) 15 (71.4)	0.253	Ref 3.41 (1.22 to 9.55)	Ref 1.72 (0.44 to 6.67)
Alcohol use (n, %)					
No Yes	53 (57.0) 9 (36.0)	40 (43.0) 16 (64.0)	0.066	Ref 2.36 (0.94 to 5.88)	Ref 2.88 (0.93 to 8.91)
Contact with TB¶ case (n, %)					
No Yes	51 (57.3) 11 (37.9)	38 (42.7) 18 (62.1)	0.367	Ref 2.20 (0.93 to 5.19)	Ref 1.69 (0.54 to 5.33)
Clinical and laboratory characteristics					
Duration of ART use (n, %)					
≤3 years >3 years	45 (61.6) 17 (37.8)	28 (38.4) 28 (62.2)	Ref **0.013**	Ref 2.65 (1.23 to 5.69)	Ref 3.99 (1.55 to 10.28)

The bold values are factors meeting statistical significance at p<0.05.

*Negative QFT-Plus test

†Positive & indeterminate QFT-Plus test

‡crude OR

§adjusted OR

¶Tuberculosis

ART, antiretroviral therapy; BMI, body mass index; QFT-Plus, QuantiFERON-TB; TB, tuberculosis.

## Discussion

We used the QFT-Plus test to estimate the prevalence and determinants of TB infection among PLHIV in the context of high TB/HIV burden and near-universal ART and TPT access. We found the prevalence of TB infection estimated by QFT-Plus test results, comprising positive and indeterminate test results, to be 47.9%. We found that the QFT-Plus test positivity in this population of PLHIV was 39.7%, a rate corresponding to estimates from the general population in an equivalent context.^33^ This differs slightly from a similar study conducted among pregnant women in Kenya, where 35.8% of the pregnant women living with HIV had TB infection by QFT-Plus test positivity rate equivalent to those without HIV.^32^ In Nigeria, in a similar high HIV/TB burden setup, Aladesanmi *et al* found that 53% of PLHIV had TB infection.^34^ Globally, TB infection is estimated at 25% of the world population.^1 2^ This variability, both geographically and by population, underscores the need for regular context-specific determination of the prevalence of TB infection for policy-making and clinical practice.

The most significant benefit following TPT is among PLHIV with confirmed TB infection.^17^ Further, a recent review demonstrated higher proportions of PLHIV starting and completing TPT with testing for TB infection.^35^ The current WHO and local guidelines indicate that confirming TB infection is not a prerequisite for TPT among PLHIV.^9 18–20 28^ It is essential to consider the clinical and cost implications of treating individuals who may not benefit, either because they are uninfected or because of non-adherence to TPT.^17 35^ From our findings, approximately 60% of PLHIV receiving TPT may not benefit, potentially exposing them to avoidable adverse drug events, drug-drug interactions, polypharmacy and increased risk of non-adherence. Although a recent review concluded that providing TPT to PLHIV is cost-effective for preventing TB disease,^36^ only two studies assessing the incremental cost of test-directed treatment from lower-income and middle-income countries were included.^37 38^ Furthermore, these were conducted among pregnant women, and the findings may not be generalisable. This, therefore, remains an area for further investigation.

We found that indeterminate results were reported in 8.3% of the cases, a rate similar to that of the general population but lower than those reported among PLHIV.^39–41^ An altered immune response increases the likelihood of indeterminate results.^13 39 40 42–44^ In HIV, a low CD4 cell count is often implicated.^41 45 46^ However, HIV can lead to indeterminate results even with normal CD4 cell counts due to altered T-cell function.^47–49^ The rate of indeterminate result findings comparable to that of the average population in our study can be explained in two ways. First, although CD4 counts were unavailable, most participants-initiated ART early in the ‘test and treat’ era. We speculated that most had competent immunity.^50^ Second, most participants had been on ART long enough (median duration on ART being 3.9 years), and ART duration influences immune recovery.^50–53^

A QFT-Plus test result is interpreted as indeterminate when there is a decrease in IFN-γ production in the mitogen tube (positive control) or/and an increase in the Nil tube (negative control).^29^ In our study, we found decreased production of IFN-γ in the mitogen tube in 8 out of 10 cases. An altered immune response will lead to an insufficient reaction in the mitogen tube, corresponding to the incapacity of lymphocytes to secrete IFN-γ.^46 54–58^ Possibly, the T lymphocytes among the participants with indeterminate results were compromised in quality and were thus unable to produce sufficient levels of IFN-γ.^46 57^ In support, most of the participants with indeterminate results had been on ART for less than a year, all were ambulatory, and none had a known illness other than HIV. In cases of immune suppression, repeat testing is advised after recovery.^54 55^ Further, with indeterminate results, TB infection cannot be ruled out with certainty, and the prognosis may be poorer.^54 55^ The increased response in the Nil tube found in three participants may signify residual IFN-γ due to ongoing infection,^58^ and TB infection could not be ruled out.

Interpreting indeterminate results is a clinical dilemma. The WHO and local Kenyan guidelines do not require testing for TB infection before initiating TPT, and there is no guidance for handling indeterminate results where testing is done.^9 18 20 28^ Given the clinical implication and potential benefit of TPT in PLHIV,^30 31^ the possibility of immune suppression among participants with indeterminate results, the uncertainty around prognostic implications of indeterminate results^54 55^ and the resource limitation that precluded repeat testing during follow-up, the operational definition of TB infection used in the present study was justified. Indeterminate results were included in the definition of TB infection, bringing the proportion of PLHIV classified as having TB infection to 47.9%.

TB infection was significantly higher among participants who were obese/overweight (BMI≥25 kg/m^2^) and who had been on ART for over 3 years. The association with obesity differed from previous findings, although PLHIV were not explicitly considered in these studies.^59–61^ Obesity is associated with an increased risk of diabetes,^61^ which increases the risk of TB infection and progression to active TB.^62 63^ In addition, ART is implicated in various components of metabolic syndrome, including an increased risk of hyperglycaemia and diabetes.^41^ However, our study only assessed self-reported diabetes and did not measure the haemoglobin A1c level to determine its association with TB infection. We hypothesised that being on ART for over 3 years resulted in CD4 recovery to at least the lower end of the normal range with increased yield from QFT-Plus testing.^46^ There was a non-significant association of viral load with ART duration. Since the viral load was not done at the point of QFT-Plus testing, it was unsuitable for assessing immune reconstitution, explaining our findings. Although the age category was not associated with TB infection,^14 64 65^ those on ART longer were also older. Longer cumulative exposure time expected with increasing age could be a source of residual confounding. Compared with those without TB infection, those on ART longer had higher odds of being indeterminate. This is contrary to what we would expect; however, the study was not sufficiently powered to assess this association, given that the numbers with indeterminate results were small.

Alcohol use increases the likelihood of TB infection and disease.^66 67^ Although not significantly associated with TB infection on multivariable analysis, there were increased odds of TB infection with a trend towards significance. Structurally, people who consume alcohol are more likely to congregate in crowded places with high TB transmission rates. Alcohol is also known to cause immune suppression, which increases the risk of infection.^67^ There was no association between TB infection and living in a crowded place or contact with a person with TB compared with findings from other studies.^33^ This could be due to the small sample size and the reduced power required to detect an association. In addition, household crowding and the history of contact with a TB case were measured by self-report. This is subjective and disposed to social desirability and recall bias.

### Study strengths and limitations

Our study had several strengths. The involvement of participants visiting the national referral hospital, a cosmopolitan group, increased our ability to generalise our findings. We excluded participants with prior exposure to TPT, thus reducing the chance of outcome misclassification. We considered the clinical implications of indeterminate QFT-Plus results for a resource-limited setup. We confirmed the robustness of our results by performing a sensitivity analysis, assuming indeterminate results were negative. A limitation of our study is that we did not collect data on CD4 counts and could not account for the association of immune status with TB infection. This was due to changes in the guidelines, precluding the use of CD4 for HIV monitoring. Since the viral load was not done at the point of QFT-Plus testing, there was a misclassification bias. To address this limitation, we used years of ART as a proxy for immune recovery and competency.

Second, alcohol use, cigarette use, household crowding, history of contact with a TB case and presence of other chronic conditions, including autoimmune diseases, were based on self-reporting. This is subject to error and social desirability, and recall bias. Third, obesity was not correlated with other metabolic dysfunctions, and diabetes status was not objectively ascertained. Finally, this was a cross-sectional study; no long-term follow-up was conducted to see those who developed TB disease. A larger sample size would have had more power to detect associations. Therefore, studies with larger sample sizes are necessary to elucidate better the factors affecting indeterminate QFT-plus tests.

## Conclusion and recommendation

The prevalence of TB infection in this population of PLHIV who had not received TPT is higher than the global estimate, depicting high levels of exposure and better performance of the QFT-Plus test with early and longer ART use. The rates of indeterminate results were the same as those reported for the general population, further supporting the effect of ART on immunity and QFT-Plus test sensitivity. Given the existing evidence, it is important to consider the clinical and cost implications of test-directed TPT for PLHIV in low-income and middle-income countries. This will better inform policies towards recommending test-directed TPT in PLHIV never exposed to TPT. Clear directives on handling indeterminate QFT-Plus test results in PLHIV are needed. People with vulnerabilities, such as obesity and alcoholism, need to be targeted for TPT. A more in-depth analysis of the determinants of TB infection using a larger sample size is recommended.

## Data Availability

Data are available on reasonable request. All data generated or analysed during this study are not publicly available due to privacy policy regulations but are available from the corresponding author on reasonable request.
