# Supplementary material for: Prevalence and predictors of tuberculosis infection among people living with HIV in a high tuberculosis burden context
Source: BMJ Open Respir Res. 2023 May 17;10(1):e001581. doi: 10.1136/bmjresp-2022-001581 (PMC10193057; doi:10.1136/bmjresp-2022-001581)
Supplement: Supplementary data [file bmjresp-2022-001581supp001.pdf]

Supplementary Table 1 Sub-analysis to assess the association of ART duration with age, viral load level and QFT-Plus test result (n 118)

Variable	On ART <3 years (n = 73)	On ART ≥3 years (n = 45)	P-value	OR[1] (95% CI.)	Pearson chi2
Age groups (n, %)					
< 30	20 (80.0)	5 (20.0)	Ref	Ref	
30–39	25 (75.8)	8 (24.2)	0.704	1.28 (0.36-4.58)	
> 40	28 (46.7)	32 (53.3)	**0.005**	4.57 (1.42-14.7)	12.06
Viral load level (copies/ml; n, %)[2]					
≤ 40	61 (61.0)	39 (39.0)	Ref	Ref	
> 40	11 (64.7)	6 (35.3)	0.772	0.85 (0.29-2.51)	0.08
QFT-Plus test (n, %)					
Negative	45 (72.6)	17 (27.4)	Ref	Ref	
Positive	22 (47.8)	24 (52.2)	**0.009**	2.89 (1.25-6.65)	6.81
Indeterminate	6 (60.0)	4 (40.0)	0.420	1.76 (0.44-7.15)	0.65

[1]Odds ratio; [2] n=117) One participant did not have a valid viral load report; ART antiretroviral therapy
