# Supplementary material for: Prevalence and predictors of tuberculosis infection among people living with HIV in a high tuberculosis burden context
Source: BMJ Open Respir Res. 2023 May 17;10(1):e001581. doi: 10.1136/bmjresp-2022-001581 (PMC10193057; doi:10.1136/bmjresp-2022-001581)
Supplement: Supplementary data [file bmjresp-2022-001581supp002.pdf]

Supplementary Table 2 Factors associated with TB infection: Sensitivity analysis excluding category with BMI < 18.5 (n 116)

Variable	No TB infection[1] (n = 60)	TB infection[2] (n = 56)	Bivariate analysis		Multivariate analysis[8]	
			cOR[3] (95% CI.)	P- value	aOR[4] (95% CI.)	P- value
**Baseline characteristics**						
Gender (n, %)						
Male	15 (50.0)	15 (50.0)	Ref			
Female	45 (52.3)	41 (47.7)	0.91(0.40–2.10)	0.827	-	-
Age (mean, SD)	(37.8, 11.2)	(38.9, 10.4)	-	0.711	-	-
Age groups (n, %)						
< 30	15 (57.7)	11 (42.3)	Ref			
30–39	18 (60.0)	12 (40.0)	0.91 (0.31 – 2.67)	0.862		
≥ 40	27 (45.0)	33 (55.0)	1.67 (0.65– 4.27)	0.282	-	-
BMI (n, %)						
18.5 - < 25	37 (61.7)	23 (38.3)	Ref		Ref	
≥ 25.0	23 (41.1)	33 (58.9)	2.31 (1.08–4.96)	**0.027**	2.85 (1.19–6.83)	**0.018**
**Social risk factors**						
History of smoking (n, %)						
No	55 (56.7)	42 (43.3)	Ref		Ref	
Yes	5 (26.3)	14 (73.7)	3.67 (1.18– 11.4)	**0.015**	1.51 (0.36–6.34)	0.571
Alcohol use (n, %)						
No	52 (55.9)	41 (44.1)	Ref		Ref	
Yes	8 (34.8)	15 (65.2)	2.38 (0.90 –6.27	**0.069**	3.59 (1.09–11.8)	**0.035**
Household crowding (n, %)						
Not crowded	44 (52.4)	40 (47.6)	Ref			
Crowded	16 (50.0)	16 (50.0)	1.10 (0.49 –2.49)	0.819	-	-
Contact with TB[5] case (n, %)						
No	49 (56.3)	38 (43.7)	Ref		Ref	
Yes	11 (37.9)	18 (62.1)	2.11 (0.88–5.07)	**0.086**	1.63 (0.50–5.27)	0.418
**Clinical and laboratory characteristics**						
Diabetes status (n, %)						
No	57 (50.4)	56 (49.6)	Ref			
Yes	3 (100.0)	0 (0.0)	0.00(-)	0.090	-	-
Duration of ART use (n, %)[6]						
≤ 3 years	44 (62.9)	26 (37.1)	Ref		Ref	
> 3 years	15 (34.9)	28 (65.1)	3.16 (1.38–7.23)	**0.004**	3.65 (1.52– 8.73)	**0.004**
Viral load level (copies/ml; n, %)[7]						
≤ 40	51 (51.5)	48 (48.5)	Ref			
> 40	7 (46.7)	8 (53.3)	1.21 (0.41–3.62)	0.728	-	-

[1]Negative QFT-Plus test; [2]Positive & indeterminate QFT-Plus test; [3]crude odds ratio; [4]adjusted odds ratio; [5]Tuberculosis; [6] n = 113, 3 participants did not have a documented antiretroviral therapy (ART) start date; [7]n = 114, 2 participants did not have a valid viral load report: [8]Complete case analysis n=113
