# Supplementary material for: Prevalence and predictors of tuberculosis infection among people living with HIV in a high tuberculosis burden context
Source: BMJ Open Respir Res. 2023 May 17;10(1):e001581. doi: 10.1136/bmjresp-2022-001581 (PMC10193057; doi:10.1136/bmjresp-2022-001581)
Supplement: Supplementary data [file bmjresp-2022-001581supp003.pdf]

Supplementary Table 3 Factors associated with TB infection: Sensitivity analysis with indeterminate QFT-Plus test as negative (n 121)

Variable	No TB infection[1] (n = 73)	TB infection[2] (n = 48)	Bivariate analysis		Multivariate analysis[8]	
			cOR[3] (95% CI.)	P-value	aOR4 (95% CI.)	P-value
**Baseline characteristics**						
Gender (n, %)						
Male	20 (64.5)	11 (35.5)	Ref			
Female	53 (58.9)	37 (41.1)	1.27 (0.54–2.97)	0.582	-	-
Age (mean, SD)	(37.7, 11.2)	(39.5, 10.1)	-	0.807		-
Age groups (n, %)						
< 30	17 (65.4)	9 (34.6)	Ref	Ref		
30–39	23 (69.7)	10 (30.3)	0.82 (0.27 – 2.48)	0.727		
≥ 40	33 (53.2)	29 (46.8)	1.65 (0.63– 4.33)	0.296	-	-
BMI (n, %)						
< 25	46 (70.8)	19 (29.2)	Ref		Ref	
≥ 25.0	27 (48.2)	29 (51.8)	2.60 (1.20–5.63)	**0.012**	2.54 (1.14 –5.65)	**0.022**
**Social risk factors**						
History of smoking (n, %)						
No	64 (64.7)	35 (35.4)	Ref		Ref	
Yes	9 (40.9)	13 (59.1)	2.64 (1.01–6.94)	**0.040**	2.71 (0.98– 7.53)	0.055
Alcohol use (n, %)						
No	60 (62.5)	36 (37.5)	Ref			
Yes	13 (52.0)	12 (48.0)	1.53 (0.62-3.76)	0.341	-	-
Household crowding (n, %)						
Not crowded	53 (60.2)	35 (39.8)	Ref			
Crowded	20 (60.6)	13 (39.4)	0.98 (0.43 –2.23)	0.970	-	-
Contact with TB[5] case (n, %)						
No	55 (61.8)	34 (38.2)	Ref			
Yes	18 (56.3)	14 (43.8)	1.26 (0.55– 2.86)	**0.583**	-	-
**Clinical and laboratory characteristics**						
Diabetes status (n, %)						
No	70 (59.3)	48 (40.7)	Ref			
Yes	3 (100.0)	0 (0.0)	0.00(-)	0.157	-	-
Duration of ART use (n, %)[6]						
≤ 3 years	51 (69.9)	22 (30.1)	Ref		Ref	
> 3 years	21 (46.7)	24 (53.3)	2.65 (1.20–5.87)	**0.013**	2.41 (1.08– 5.37)	**0.032**
Viral load level (copies/ml; n, %)[7]						
≤ 40	61 (59.8)	41 (40.2)	Ref			
> 40	10 (58.8)	7 (41.2)	1.04 (0.37–2.97)	0.939	-	-

[1]Negative & indeterminate QFT-Plus test; [2] Positive QFT-Plus test; [3]crude odds ratio; [4]adjusted odds ratio; [5]Tuberculosis; [6] n = 118, 3 participants did not have a documented antiretroviral therapy (ART) start date; [7] n = 119, 2 participants did not have a valid viral load report; [8]Complete case analysis n=118
